# Impact of mismatch-repair deficiency on the colorectal cancer immune microenvironment

**DOI:** 10.18632/oncotarget.20241

**Published:** 2017-08-14

**Authors:** Yingyi Zhang, Zhao Sun, Xinxin Mao, Huanwen Wu, Fei Luo, Xi Wu, Liangrui Zhou, Jing Qin, Lin Zhao, Chunmei Bai

**Affiliations:** ^1^ Department of Oncology, Peking Union Medical College Hospital, Chinese Academy of Medical Sciences and Peking Union Medical College, Beijing, People’s Republic of China; ^2^ Department of Pathology, Peking Union Medical College Hospital, Chinese Academy of Medical Sciences and Peking Union Medical College, Beijing, People’s Republic of China

**Keywords:** colorectal cancer, dMMR, tumor immune microenvironment, PD-1/PD-L1

## Abstract

Colorectal cancer patients respond inconsistently to immunotherapies, likely due to the immune microenvironments around their tumors. We analyzed the relationship between deficient mismatch repair (dMMR) and the colorectal cancer immune microenvironment to identify predictors of effective immunotherapy. Colorectal cancer patients (n=113) who had undergone surgical resection were divided into dMMR and proficient mismatch repair (pMMR) groups. The levels of immune checkpoint proteins, including programmed cell death 1 (PD-1), programmed cell death 1 ligand 1 (PD-L1), indoleamine 2,3 dioxygenase and CD8 were assessed immunohistochemically. The percentage of tumor-infiltrating lymphocytes strongly positive for PD-1 (score=3) was higher in the dMMR than pMMR group (79.3% vs. 41.7%; p=0.003). The groups showed similar tumor cell PD-L1 positivity rates (34.5% vs. 35.7%, p=0.905) and PD-L1 intensity levels on immune cell infiltrates (86.2% vs. 84.5%, p=0.964). However, when a cut-off value of 80% was used for PD-L1 positivity, the rate of PD-L1 positivity on immune cell infiltrates differed between the groups (51.7% vs. 22.6%, p=0.003). The rate of high indoleamine 2,3 dioxygenase expression was greater in the dMMR than pMMR group (55.2% vs. 36.9%, p=0.026). CD8+ T cells were elevated in the dMMR group in both compartments (p=0.017 for tumor-infiltrating lymphocytes and stroma; p=0.038 for invasive front). Thus the immune microenvironment of dMMR colorectal cancer differs from that of pMMR colorectal cancer.

## INTRODUCTION

Colorectal cancer (CRC) is the third most commonly diagnosed cancer in males and the second in females worldwide [1]. In China, CRC was the fifth most commonly diagnosed cancer in males and the fourth in females in 2015, and was the fifth leading cause of cancer death in both men and women [2]. CRC should be regarded as a heterogeneous disease, as histologically identical tumors may have unique prognoses and responses to therapy [3].

Microsatellite instability (MSI-H) CRC is well-known as a special tumor subtype [4]. The mutation or hypermethylation of mismatch repair genes (MutL Homolog 1 [MLH1], MutS Homolog 2 [MSH2], MSH6, or PMS2) leads to deficient mismatch repair (dMMR) and causes error accumulation in DNA sequences, thus increasing the risk of CRC and other epithelial cancers [5]. Colon cancer patients with MSI-H often present with larger, more proximal tumors, poorer differentiation, greater mucin secretion, more lymphocytic infiltrates, and better overall survival compared to those with MSI-L/MSS (despite some negative prognostic characteristics) [6]. The promising outcomes of clinical studies (KEYNOTE016, Checkmate142) have also demonstrated that dMMR is a significant predictor of immunotherapy effectiveness. The hypothesis that dMMR tumors stimulate the immune system has been supported by observations of dense immune infiltration and a Th1-associated-cytokine-rich environment in dMMR tumors [7].

Immune checkpoints form an intricate system of inhibitory signals, and are crucial for maintaining peripheral immune tolerance and preventing autoimmunity [8]. The T-cell response depends on the balance between co-stimulatory and inhibitory signals. In the presence of activated T cells, tumor cells upregulate the expression of immunosuppression proteins, thus inhibiting the T-helper cell response and causing “T-cell exhaustion” [9]. As tumors often co-opt immune checkpoints to escape immune surveillance, inhibitors of immune checkpoints can be used to revive tumor immunity [8].

The tumor immune microenvironment is reflected by the expression of specific molecules. Programmed cell death 1 ligand 1 (PD-L1) is a critical immune checkpoint molecule that is induced by pro-inflammatory cytokines, especially type II interferon, in an important self-limiting mechanism to prevent rampant autoimmunity. PD-L1 is also upregulated on tumor cells and tumor-associated myeloid cells, and impairs T-cell-induced immune responses upon engaging its cognate co-inhibitory receptor, programmed cell death 1 (PD-1), which is always highly expressed on tumor-infiltrating lymphocytes (TILs) [10]. In addition, indoleamine 2,3 dioxygenase (IDO), a tryptophan-catabolizing enzyme, was recently discovered to be induced in an immune escape mechanism for tumor cells. Tryptophan is essential for T-cell function, so when IDO is upregulated, the depletion of tryptophan causes T-cell apoptosis and ultimately promotes cancer progression by suppressing T-cell immunity. Thus, IDO is a potential target for anticancer therapy [11].

In the tumor microenvironment and regional lymph nodes, the presence of TILs—especially CD8+ lymphocytes—has been linked to a better prognosis [8]. However, Llosa and colleagues recently refined these classic observations by demonstrating that several immune checkpoint ligands were upregulated in the dMMR tumor microenvironment, including PD-1, PD-L1, cytotoxic T-lymphocyte associated protein 4 (CTLA-4), lymphocyte-activation gene 3 (LAG-3) and IDO. Thus, the active immune microenvironment appears to be counterbalanced by immune inhibitory signals that prevent tumor elimination [12]. PD-1 and CTLA-4 are critically involved in immune checkpoints, and potential tumor-reactive lymphocytes are often restrained by CTLA- and/or PD-1-transduced signals, reflecting the ability of many cancers to upregulate the corresponding ligands [13].

Approximately 15% of CRC cases exhibit dMMR. In advanced dMMR CRC patients, anti-PD-1 monoclonal antibodies have displayed promising therapeutic effects. Thus, we considered it clinically significant to research the specific features of the immune microenvironment in patients with dMMR CRC, as we have done in the present study.

## RESULTS

### Patient characteristics

Based on our inclusion criteria, we selected a total of 205 patients, among whom there were 29 dMMR patients and 176 pMMR patients. Using a 1:3 matching method (with sex, age, location and TNM stage as the matching criteria), we selected 87 pMMR patients. Paraffin blocks of tumor tissues were missing from three of these 87 patients; thus, we included 113 patients in our study (29 dMMR patients and 84 pMMR patients).

**Figure 1:**
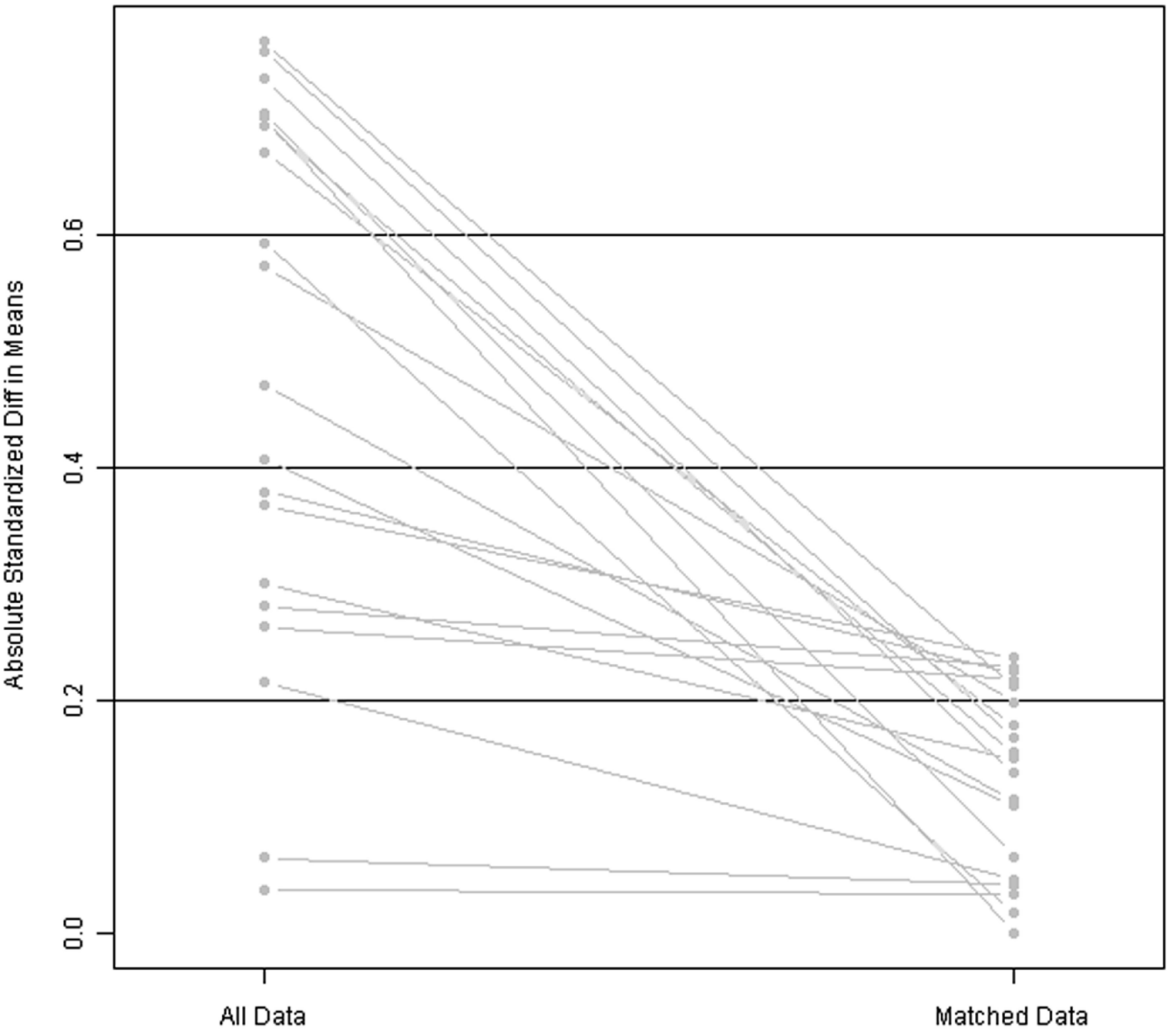
Line plot of standardized differences before and after matching

As a result of the 1:3 matching method, confounding bias was reduced: the relative multivariate imbalance was 0.609 after the matching, versus 0.786 before the matching. A line plot of the standardized differences before and after matching is shown in Figure 1. The patients’ clinical and pathological parameters are shown in Table 1.

**Table 1:** Patients’ clinical and pathological parameters (n=113)

		dMMR(n=29)	pMMR(n=84)	p value
sex	male	13(44.8%)	40(47.6%)	0.795
	female	16(55.2%)	44 (52.4%)	
age	<65y	16(55.2%)	45(53.6%)	0.881
	≥65y	13(44.8%)	39(46.4%)	
tumor location	right	23 (79.3%)	68 (81.0%)	0.327
	left	5 (17.2%)	16 (19.0%)	
	whole	1 (3.4%)	0 (0.0%)	
tumor size	≤5cm	11 (37.9%)	54 (64.3%)	0.013
	>5cm	18 (62.1%)	30 (35.7%)	
differentiation degree	poor	3 (10.3%)	8(9.5%)	0.940
	mediate	20 (69.0%)	61(72.6%)	
	high	6 (20.7%)	15(17.9%)	
histologic subtype	conventional adenocarcinoma	23 (79.3%)	74 (88.1%)	0.105
	mucinous	0 (0.0%)	4 (4.8%)	
	mixed	6 (20.7%)	6 (7.1%)	
primary TNM stage	I	1 (3.4%)	9 (10.7%)	0.247
	II	22 (75.9%)	48 (57.1%)	
	III	6 (20.7%)	27 (32.1%)	
involved lymph node number	0	22 (75.9%)	61 (72.6%)	0.192
	1-3	7 (24.1%)	15 (17.9%)	
	≥4	0 (0.0%)	8 (9.5%)	
vascular invasion	yes	3(10.3%)	14(16.7%)	0.553
	no	26(89.7%)	70(83.3%)	

### Immunohistochemical (IHC) expression of immune checkpoint proteins in dMMR and pMMR patients

#### PD-1 expression of TILs

**Figure 2:**
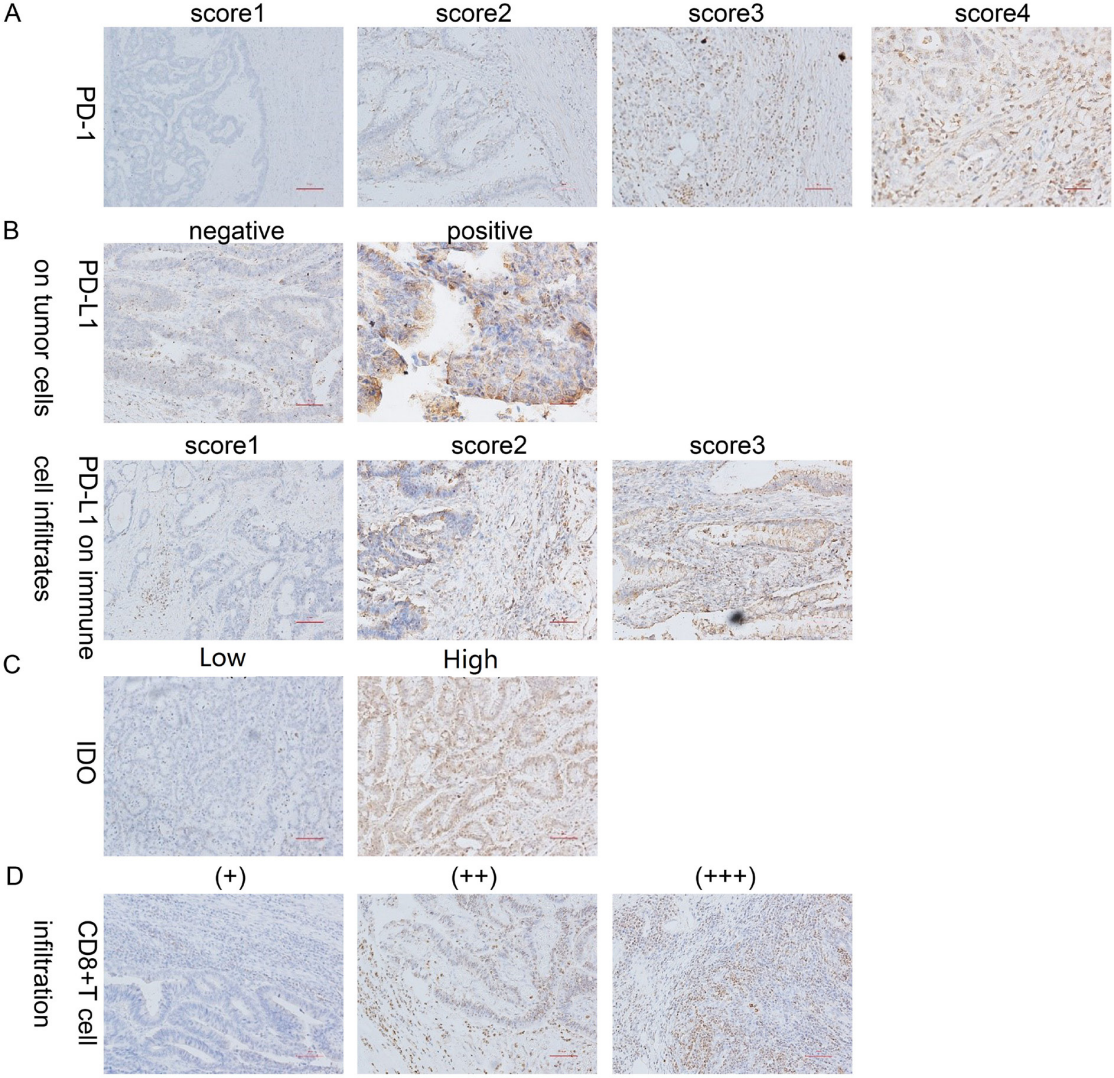
IHC staining for PD-1, PD-L1, CD8 and IDO expression **(A)** Representative patterns of PD-1 expression from TILs with scores of 0, 1, 2 and 3 are shown. **(B)** Representative patterns of PD-L1 expression from negative and positive tumor cells are shown, as well as PD-L1 expression from immune cell infiltrates with scores of 1, 2 and 3. **(C)** Representative patterns of IDO expression from tumor cells with low and high expression are shown. **(D)** Representative patterns of CD8 expression from TILs with (+), (++) and (+++) expression are shown. Original magnification, ×100 (scale bars, 100 mm).

**Figure 3:**
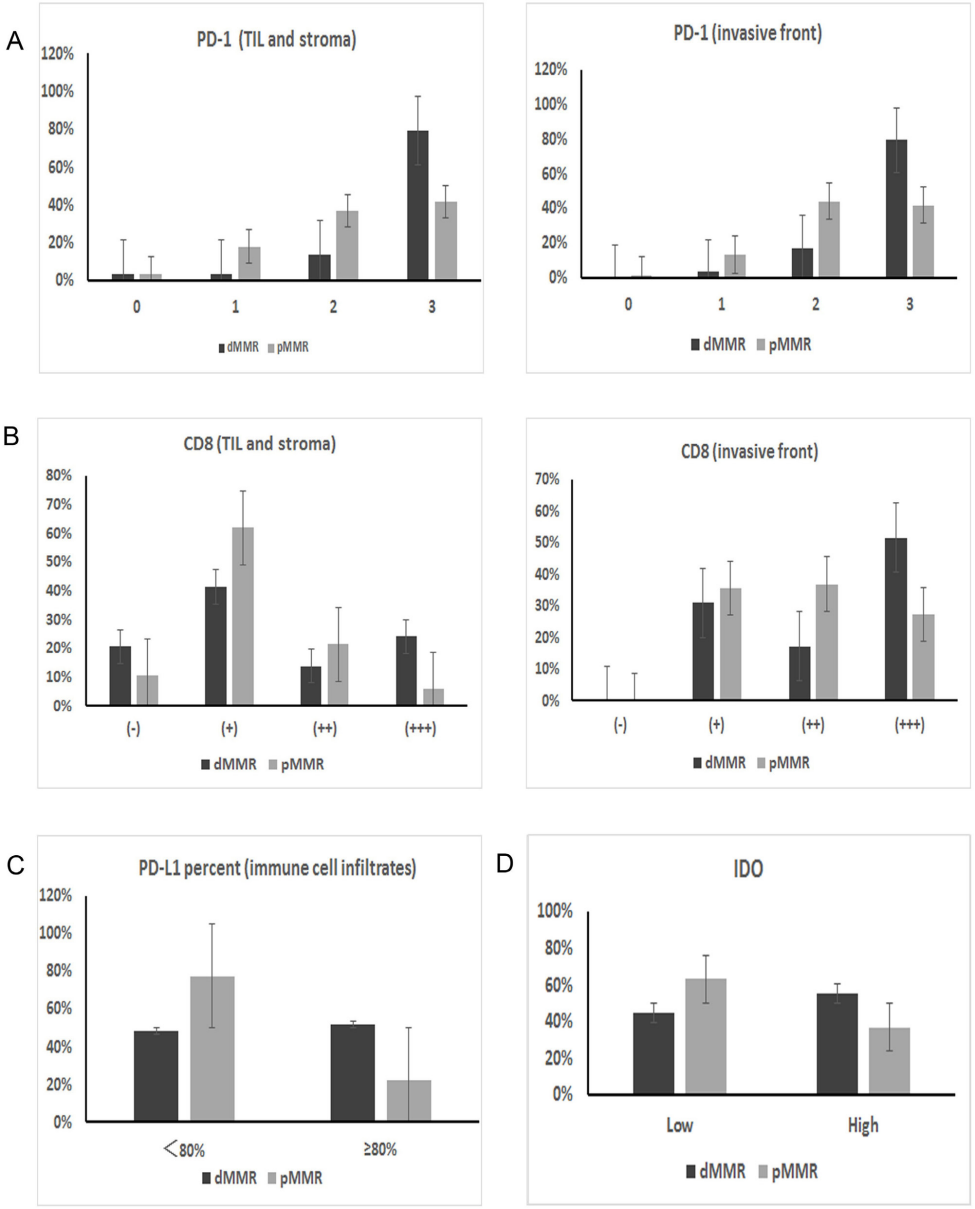
IHC expression differences in immune checkpoint proteins between dMMR and pMMR patients **(A)** PD-1 expression on TILs from the TIL and stromal compartment and the invasive front compartment was significantly higher in dMMR tumors than in pMMR tumors (*p=0.003* for both compartments). **(B)** Similarly, the number of CD8+ T cells in the two compartments was also higher in dMMR tumors than in pMMR tumors (*p=0.017* for TIL and stroma; *p=0.038* for invasive front). **(C)** PD-L1 expression on immune cell infiltrates; the expression percentage differed significantly between the two groups when the cut-off value was set as 80% (*p=0.003*). **(D)** IDO expression in tumor cells also differed significantly between dMMR and pMMR tumors (*p=0.026*).

**Figure 4:**
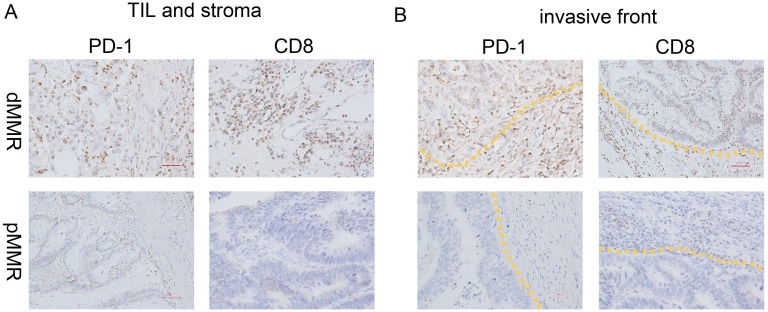
PD-1 and CD8 expression of dMMR and pMMR CRC specimens IHC analysis of PD-1 and CD8 expression in **(A)** the TIL and stromal compartment and **(B)** the invasive front compartment was performed on formalin-fixed, paraffin-embedded tissue sections of a representative set of dMMR (top) and pMMR (bottom) CRC specimens. Original magnification, ×200 (scale bars, 50 mm).

The intensity of PD-1 expression varied, and the protein was predominantly immunolocalized to the membrane and cytoplasm of TILs (Figure 2A). As shown in Table 2, the percentage of cells with strongly positive PD-1 expression (score=3) was significantly higher in the dMMR group than in the pMMR group (79.3% vs. 41.7%, p=0.003) (Figure 3A, Figure 4).

**Table 2:** IHC scores of dMMR and pMMR patients

	IHC score	dMMR	pMMR	p
PD-1 (TIL and stroma)	0	1 (3.4%)	3 (3.6%)	0.003**
	1	1 (3.4%)	15 (17.9%)	
	2	4 (13.8%)	31 (36.9%)	
	3	23 (79.3%)	35 (41.7%)	
PD-1 (invasive front)	0	0 (0%)	1 (1.2%)	0.003**
	1	1 (3.4%)	11 (13.1%)	
	2	5 (17.2%)	37 (44.0%)	
	3	23 (79.3%)	35 (41.7%)	
PD-L1(tumor cells)	(-)	19 (65.5%)	54 (64.3%)	0.905
	(+)	10 (34.5%)	30 (35.7%)	
PD-L1 percent(immune cell infiltrates)	<60%	9 (31.0%)	35 (41.7%)	0.311
	≥60%	20 (69.0%)	49 (58.3%)	
	<70%	11 (37.9%)	48 (57.1%)	0.074
	≥70%	18 (62.1%)	36 (42.9%)	
	<80%	14 (48.3%)	65 (77.4%)	0.003**
	≥80%	15 (51.7%)	19 (22.6%)	
PD-L1 intensity(immune cell infiltrates)	0	0 (0%)	1 (1.2%)	0.964
	1	4 (13.8%)	12 (14.3%)	
	2	19 (65.5%)	51 (60.7%)	
	3	6 (20.7%)	20 (23.8%)	
AIS	0-50	4 (13.8%)	11 (13.1%)	0.155
	60-100	6 (20.7%)	23 (27.4%)	
	110-200	13 (44.8%)	41 (48.9%)	
	210-300	6 (20.7%)	9 (10.7%)	
IDO	low	13 (44.8%)	53 (63.1%)	0.026*
	high	16 (55.2%)	31 (36.9%)	
CD8 (TIL and stroma)	(-)	6 (20.7%)	9 (10.7%)	0.017*
	(+)	12 (41.4%)	52 (61.9%)	
	(++)	4 (13.8%)	18 (21.4%)	
	(+++)	7 (24.1%)	5 (6.0%)	
CD8 (invasive front)	(-)	0 (0%)	0 (0%)	0.038*
	(+)	9 (31%)	30 (35.7%)	
	(++)	5 (17.2%)	31 (36.9%)	
	(+++)	15 (51.7%)	23 (27.4%)	

#### PD-L1 expression on tumor cells and immune cell infiltrates

PD-L1 was predominantly immunolocalized to the membrane and cytoplasm of tumor cells and immune cell infiltrates (tissue macrophages, dendritic cells and Langerhans cells) (Figure 2B). On tumor cells, the total PD-L1 positivity rate was 35.4%. There was no significant difference in PD-L1 positivity between the dMMR and pMMR groups (34.5% of the dMMR group vs. 35.7% of the pMMR group, p=0.905). As for immune cell infiltrates, 85.0% of cells expressed PD-L1 at a high intensity (score=2/3), and there was no significant difference in the expression intensity between the dMMR and pMMR groups (86.2% of the dMMR group vs. 84.5% of the pMMR group, p=0.964). On the other hand, when we set the cut-off value for PD-L1 positivity as 80%, the expression percentage of PD-L1 on immune cell infiltrates differed significantly between the groups (51.7% of the dMMR group vs. 22.6% of the pMMR group, p=0.003) (Figure 3C). Interestingly, the adjusted inflammation scores were very similar for dMMR and pMMR tumors (Table 2).

To verify the rationality of setting 80% positive PD-L1 expression on immune cell infiltrates as the cut-off value for dMMR CRC, we generated a receiver operating characteristic curve and determined the area under the curve (AUC) when a cut-off value of 60%, 70% or 80% positive immune cell infiltrates was used. The 80% cut-off value had the best sensitivity (77.38±10.36 for 80% vs. 41.67±10.67 for 60% and 57.14±11.24 for 70%) and the largest AUC (0.646 for 80% vs. 0.553 for 60% and 0.596 for 70%), although it had the lowest specificity (51.72±19.22 for 80% vs. 68.97±19.77 for 60% and 62.07±19.77 for 70%) (Supplementary Figure 1).

#### IDO expression of tumor cells

IDO was predominantly immunolocalized to the cytoplasm of tumor cells (Figure 2C). The percentage of cells with high IDO expression was 41.6% overall, and was higher in dMMR CRC than in pMMR CRC (55.2% of the dMMR group vs. 36.9% of the pMMR group, p=0.026) (Figure 3D).

#### CD8 expression of TILs

CD8 was predominantly immunolocalized to the cytoplasm of TILs (Figure 2D). The percentage of cells with high CD8 expression (++/+++) was 30.1% in the TIL and stromal compartment and 65.5% in the invasive front compartment. As shown in Table 2, the number of CD8+ T cells was greater in the dMMR group than in the pMMR group in both compartments (p=0.017 for TIL and stroma; p=0.038 for invasive front) (Figure 3B, Figure 4).

### IHC score comparison by the Mann-Whitney U test

We then used the Mann-Whitney U test to determine the correlation between the MMR protein expression status and the IHC scores of the immune checkpoint proteins. This confirmed that PD-1 expression on immune cell infiltrates in the TIL and stromal compartment and the invasive front compartment differed significantly between the dMMR and pMMR CRC groups (p=0.001 for both), as did the PD-L1 expression percentage on immune cell infiltrates (p=0.036) and IDO expression (p=0.014). However, CD8 expression did not differ significantly between the groups (Table 3).

**Table 3:** IHC score comparison analysis with the Mann-Whitney U test

	U value	p value
PD-1 (TIL and stroma)	760.5	0.001^**^
PD-1 (invasive front)	747.5	0.001^**^
PD-L1(tumor cells)	1198	0.874
PD-L1 intensity(immune cell infiltrates)	1203	0.912
PD-L1 percent(immune cell infiltrates)	903	0.036^*^
PD-L1(AIS)	1015.5	0.181
IDO	1051.5	0.014*
CD8 (TIL and stroma)	1138.5	0.561
CD8 (invasive front)	986	0.106

**Figure 5:**
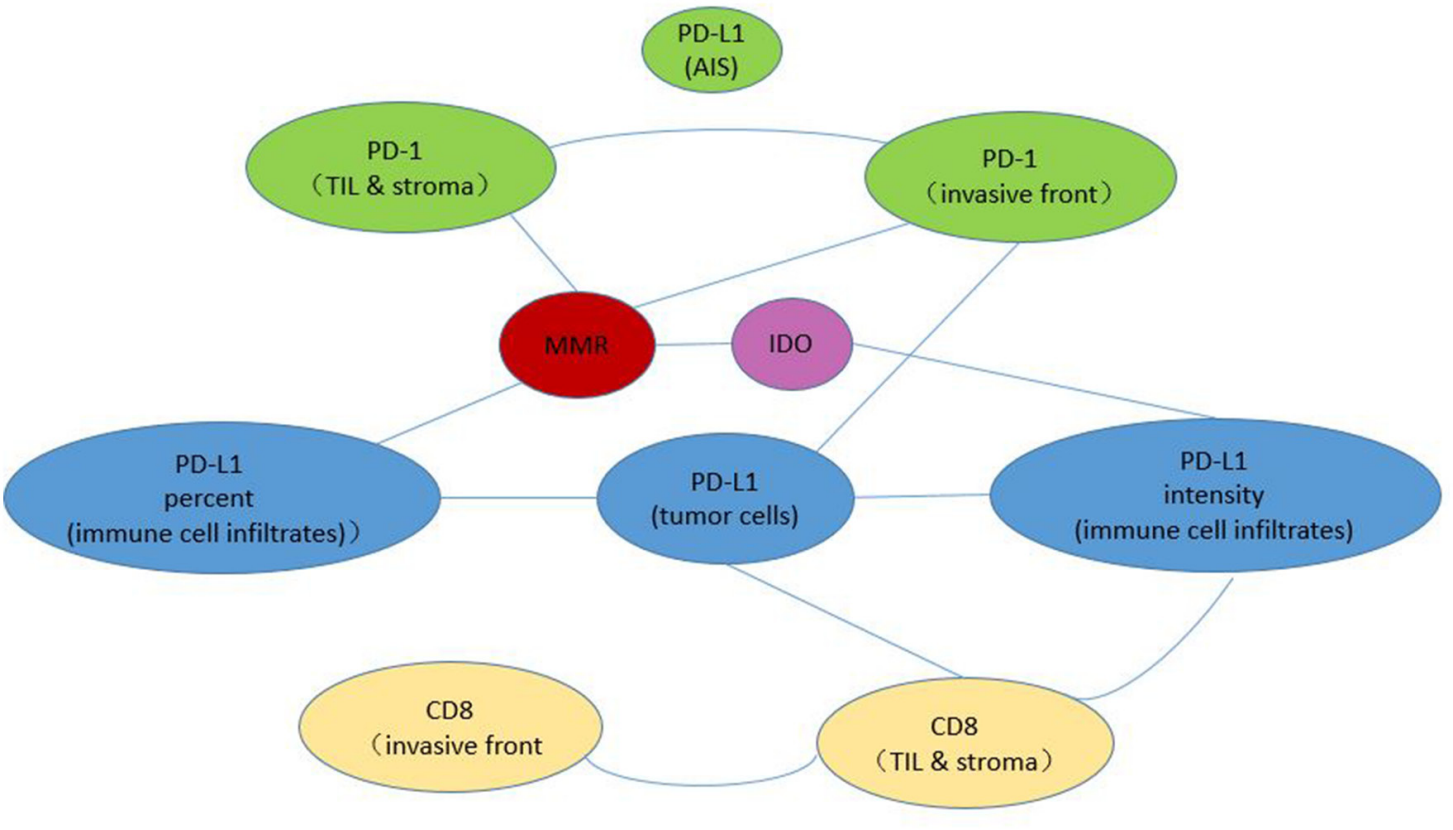
Correlation analysis with the Mann-Whitney U test Each line between two circles denotes a statistically significant correlation between these two IHC parameters.

We also used the Mann-Whitney U test to analyze the correlations among the IHC scores of the immune checkpoint proteins. As is shown in Figure 5, MMR status was signicifantly corelated with PD-1 expression, PD-L1 expression on immune cell infiltrates and IDO expression (p<0.05). PD-L1 expression on tumor cells was signicifantly corelated with PD-L1 expression on immune cell infiltrates and PD-1 expression in invasive front compartment (p<0.05).

## DISCUSSION

In this study, we used IHC to detect patients’ MMR status. IHC has been shown to have comparable performance to and high concordance with MSI testing by fluorescent multiplex PCR [14].

Our results indicated that in patients with dMMR, PD-1 was significantly upregulated in TILs, and the number of CD8+ TILs was elevated in both the TIL and stromal compartment and the invasive front compartment. This can be well explained by the mechanisms whereby MMR impacts the tumor microenvironment. dMMR tumors result from frameshift mutations within coding sequences, which often produce functionally inactive proteins. Major histocompatibility complex I expressed by a tumor can present these abnormal peptides to cytotoxic T-lymphocytes (CTLs) as neoantigens, thus increasing the TIL density and triggering an immune response in the host. As a result, cancer immune editing can occur in tumor microenvironment, and can be described in three phases - elimination, equilibrium, and escape - ultimately causing immune tolerance. The PD-1 pathway activates this process of immune editing, whereas immune checkpoint inhibitors can interrupt this pathway and stimulate activated T cells as well as antitumor immunity [6].

We also demonstrated that the PD-L1 expression percentage on immune cell infiltrates was higher in dMMR CRC than in pMMR CRC, while PD-L1 expression on tumor cells did not differ significantly between these two groups. Furthermore, we found that 80% might be the proper cut-off value for PD-L1 positivity on immune cell infiltrates. Similarly, Llosa and colleagues demonstrated that tumors with dMMR had high Th1/CTL infiltrates, and that tumors with MSI significantly overexpressed PD-1/PD-L1. Furthermore, in MSI CRC, PD-L1 was found to be expressed not only on tumor cells, but also on TILs and/or myeloid cells [12]. In another CRC study, PD-1 upregulation alone (induced by T-cell priming or reactivation in tumor drainage lymph nodes) had no direct effect on the functionality of activated CD8+ T cells. Only after activated PD-1+ CD8+ T cells migrated to the tumor and bound to PD-L1 were cytokines (IL-2 and IFN-γ) produced, stimulating the cytolytic activity of CD8+ T cells and finally causing the exhaustion or dysfunction of T cells [15]. Thus, we hypothesize that in the dMMR colon cancer immune microenvironment, aside from tumor cells, immune cell infiltrates also highly express PD-L1, which enhances the immune-escape effects of this pathway. Therefore, in clinical screening tests for potential benefit from anti-PD-1/L1 blockades, we suggest using the PD-L1 expression percentage of immune cell infiltrates as another important indicator, as well as PD-1 expression.

Additionally, our correlation analyses revealed that in the tumor microenvironment, although tumor cell PD-L1 expression was not related to MMR, it was related to PD-L1 and PD-1 expression in immune cell infiltrates in the invasive front compartment, and to the CD8+ T-cell number in the TIL and stromal compartment. A possible explanation is that tumor cells expressing PD-L1 trigger the immune response in both dMMR and pMMR colon tumors, but depending on the MMR status, there may be differences in the intensity of CTLs migrating to the tumor tissue, the expression of PD-L1 on immune cell infiltrates, and the extent of PD-1 upregulation. In other words, the subsequent immune response intensity after triggering depends on the MMR subtype. On the other hand, other studies have found that PD-L1-negative tumors may also respond to anti-PD-1 immune checkpoint inhibitors [16], possibly because some tumors express PD-L2 [17]. Thus, depending upon which interactions dominate in a particular cancer, PD-1 and PD-L1 antibodies might not have redundant activity. Based on this hypothesis, we will consider testing PD-L2 expression in future studies.

More significantly, it has been proposed that four different types of tumor microenvironments exist, based on the presence of CTLs and the expression of PD-L1. Researchers could take advantage of these distinct characteristics to design rational cancer therapies [18]. Based on large-scale data, a new classification system of CRC called consensus molecular subtypes has also been established [19].

In conclusion, the immune microenvironment of dMMR CRC differs from that of pMMR CRC. The immune microenvironment of dMMR CRC is characterized by the upregulation of PD-1, infiltration of CD8+ T cells, overexpression of PD-L1 on immune cell infiltrates, and upregulation of IDO.

## MATERIALS AND METHODS

### Patient selection

Patients were included in this study if they were surgically treated for primary CRC from November 2014 to December 2015 at Peking Union Medical College Hospital and had not received pre-operative chemotherapy or radiotherapy. Patients with other types of tumors at the same time were excluded. Four types of MMR proteins (MLH1, MSH2, MSH6 and PMS2) were detected by IHC. Then, patients (n=205) were divided into the dMMR group (if they were deficient in any of these four MMR proteins, n=29) and the pMMR group (if they expressed all four of the MMR proteins, n=176). All dMMR patients were included in our study. In addition, based on the dMMR patients, we performed 1:3 matching with pMMR patients according to sex, age, location and TNM stage. Available paraffin blocks of tumor tissues were obtained from the archival collections of the Department of Pathology.

### IHC staining process and antibodies

First, 3-μm thick consecutive sections were cut with a microtome, dewaxed in xylene and rehydrated through graded ethanol solutions. For antigen retrieval, the tissue sections were heated at 100°C for 30 min in an ethylenediaminetetraacetic acid solution when needed. Sections were cooled down and immersed in a 0.3% H_2_O_2_ solution for 20 min to block endogenous peroxidase activity, and then were rinsed in phosphate-buffered saline (PBS) for 5 min, blocked with 5% bovine serum albumin at room temperature for 20 min, and incubated with primary antibodies against PD-1 (diluted 1:200), PD-L1 (diluted 1:200), IDO (diluted 1:200), or CD8 (diluted 1:200) at 4°C overnight. For negative controls, the specific primary antibody was replaced with PBS. After three PBS washes, sections were incubated with secondary antibodies for 30 min at room temperature. Diaminobenzene was used as the chromogen, and hematoxylin was used as the nuclear counterstain. Sections were dehydrated, cleared and mounted.

The primary antibodies were all purchased from Protein Tech (Rosemont, Illinois, the US): the PD-1/CD279 Mouse Monoclonal Antibody (66220-1-Ig), PD-L1/CD274 Mouse Monoclonal Antibody (66248-1-Ig), CD8A Rabbit Polyclonal Antibody (17335-1-AP), and IDO1 Rabbit Polyclonal Antibody (13268-1-AP). Secondary antibodies were purchased from ZSGB-BIO (Beijing, China): the goat anti-mouse IgG kit (PV-6002) and goat anti-rabbit IgG kit (PV-6001).

### Quantification of IHC staining

The pathologic diagnosis was confirmed by one of two board-certified pathologists (X.X. Mao or H.W. Wu) who reviewed formalin-fixed, paraffin-embedded tissue sections stained with hematoxylin & eosin. A representative paraffin block from each specimen was chosen for IHC analysis. Each IHC specimen was independently quantified by two pathologists (X.X. Mao and H.W. Wu) who were blinded to patient outcomes, and differences were adjudicated.

We distinguished two compartments: (1) the TIL and stromal compartment, where TILs are lymphocytes intercalated within the glandular or medullary epithelial component of the tumor, and stroma are T cells in the tumor stroma surrounding the epithelial component of the tumor; and (2) the invasive front, where the tumor invades the lamina propria. Infiltration of these T-cell types into either compartment was quantitated numerically when PD-1 and CD8 expression were analyzed by IHC.

PD-1 expression was observed on TILs, and the proportion was scored from 0 to 3: 0 “none” (0% of lymphocytes), 1 “focal” (isolated, <5% of lymphocytes), 2 “moderate” (5-50% of lymphocytes), or 3 “severe” (>50% of lymphocytes) [20].

PD-L1 expression was observed on both tumor cells and “immune cell infiltrates,” which refers to tissue macrophages, dendritic cells and Langerhans cells. Tumor cells with 5% cytoplasmic expression of PD-L1 were considered “positive” [20]. Immune cell infiltrates in the tumor nest were counted as follows: five areas in the tumor nest with the most intense immune cell infiltration (including macrophages, dendritic cells and Langerhans cells) were selected at a low magnification (Original magnification, ×100), and were subsequently counted and recorded under a high-power field (Original magnification, ×200). The results from the five areas were averaged and used in the statistical analysis. The intensity of immune cell infiltrates was assigned a semi-quantitative score from 0 to 3: 0 “none” (no immune cell infiltrates), 1 “focal” (mostly perivascular in the tumor with some intratumoral extension), 2 “moderate” (prominent extension of immune cell infiltrates away from perivascular areas and among tumor cells), or 3 “severe” (immune cell infiltrates obscuring the tumor). An adjusted inflammation score was defined as the intensity of intratumoral inflammation multiplied by the percentage of inflammatory cells expressing PD-L1, reflecting PD-L1 in the inflammatory host response [10, 21, 22].

The staining of IDO was visualized and classified based on the percentage of positive cells (score 0: ≤5%, score 1: 6-25%, score 2: 26-50%, score 3: 51-75%, score 4: >75%) and the intensity of staining (score 0: no pigment, score 1: canary, score 2: yellow, score 3: brown). The total score was calculated as the percentage score and multiplied by the intensity score, and was graded as low for a score of 0-4 and high for a score of 5-12 [23].

Five areas in the tumor nest with the most intense infiltration of CD8+ T cells were selected at low magnification (Original magnification, ×100), and then the CD8+ T cells were counted and recorded under a high-power field (Original magnification, ×200) [24]. CD8+ T cells in either compartment were quantitated numerically and displayed as the average number of stained cells per high-power field: 0 (-), ≤50 (+), 51-100 (++), >100 (+++) [12].

### Statistical analysis

Neighboring Propensity Scores were used for the 1:3 matching method. The pathologic features of the dMMR and pMMR groups were compared by χ^2^ or Fisher’s exact tests. P values <0.05 were considered statistically significant. The mean IHC scores were compared between dMMR and pMMR patients by the nonparametric Mann-Whitney U test. Different cut-off values were assessed in terms of their effects on the sensitivity, specificity and AUC. All statistical analyses were performed with SPSS software, version 23.0 (IBM SPSS statistics 23) and MedCalc (MedCalc Software, Ostend, Belgium).

## SUPPLEMENTARY MATERIALS FIGURES AND TABLE


