# Supplementary material for: Impact of mismatch-repair deficiency on the colorectal cancer immune microenvironment
Source: Oncotarget. 2017 Aug 14;8(49):85526–36. doi: 10.18632/oncotarget.20241 (PMC5689628; doi:10.18632/oncotarget.20241)
Supplement: Supplementary file 1 [file oncotarget-08-85526-s001.pdf]

Impact of mismatch-repair deficiency on the colorectal cancer immune microenvironment

SUPPLEMENTARY MATERIALS

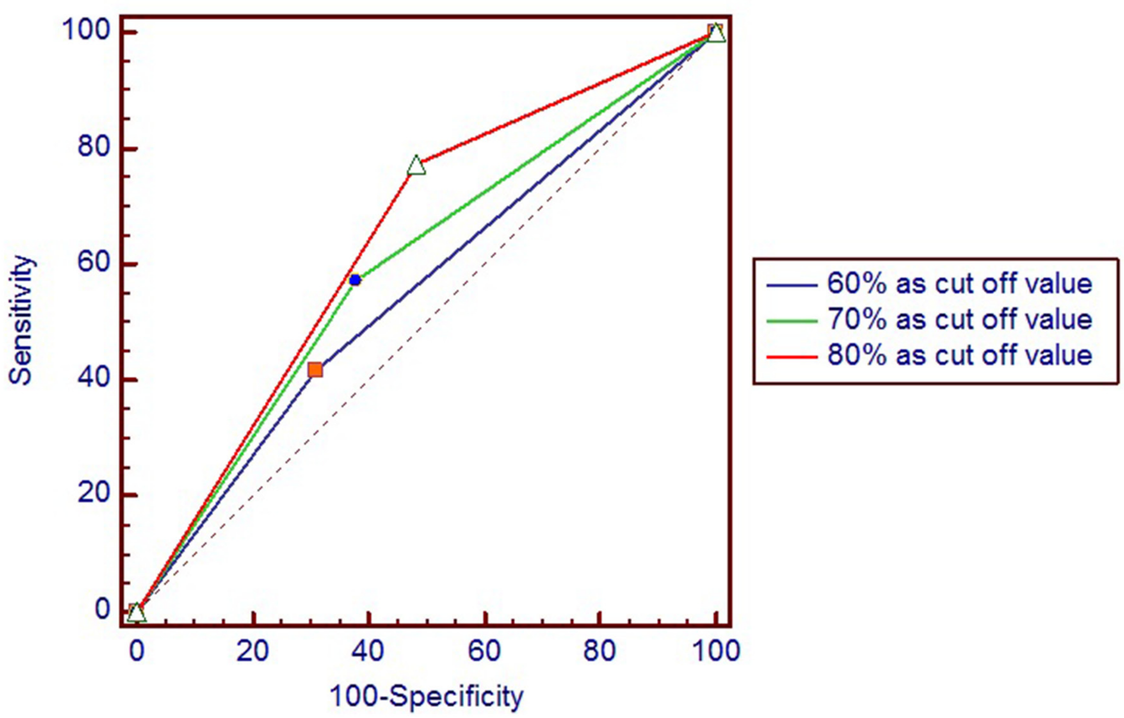

Supplementary Figure 1: Receiver operating characteristic curve to compare the AUCs with 60%, 70% and 80% positive immune cell infiltrates as cut-off values. Blue line: 60% as cut-off value, AUC= 0.553; Green line: 70% as cut-off value, AUC=0.596; Red line: 80% as cut-off value, AUC=0.646.
